# The Preventive Effect of Oxytocin on Retinopathy in Streptozotocin- Induced Diabetic Rats

**DOI:** 10.4274/tjo.galenos.2018.47897

**Published:** 2019-04-30

**Authors:** Cumali Değirmenci, Filiz Afrashi, Oytun Erbaş, Hüseyin Aktuğ, Dilek Taşkıran

**Affiliations:** 1 Ege University Faculty of Medicine, Department of Ophthalmology, İzmir, Turkey; 2 İstanbul Bilim University Faculty of Medicine, Department of Physiology, İstanbul, Turkey; 3 Ege University Faculty of Medicine, Department of Histology and Embryology, İzmir, Turkey; 4 Ege University Faculty of Medicine, Department of Physiology, İzmir, Turkey

**Keywords:** Immunohistochemistry, oxytocin, retinopathy, streptozotocin, VEGF

## Abstract

**Objectives:**

The aim of this study was to investigate the impact of intravitreal and intraperitoneal use of oxytocin (OT) on retinopathy in streptozotocin-induced diabetic rats.

**Materials and Methods:**

Twenty-four 6-8-week-old adult male and female Sprague Dawley rats were used in the study. Diabetes was induced in the rats with a single injection of intraperitoneal streptozotocin. Diabetes was verified after 48 hours by measuring blood glucose levels of 260 mg/dl (14.4 mmol/L) or higher in diabetic rats. The rats were divided into 4 groups and treated as follows: intravitreal physiological saline group (0.01 mL saline weekly), intravitreal OT group (10 μU/μL OT weekly), intraperitoneal physiological saline group (1 mL daily), and intraperitoneal OT group (100 IU/kg OT daily). Hamilton syringes fitted with 27-gauge needles were used for intraperitoneal injections while 31-gauge needles were used for intravitreal injection. After 4 weeks of treatment the rats were euthanized to evaluate outer nuclear layer (ONL) thickness, vascular endothelial growth factor (VEGF) immunoexpression, and plasma VEGF levels from blood samples obtained by cardiac puncture.

**Results:**

Morphometric analysis of retinal cross-sections showed that intravitreal and intraperitoneal OT significantly increased ONL thickness compared to physiological saline-treated groups. Also, OT treatment significantly decreased VEGF protein expression compared with the physiological saline groups. Plasma VEGF level was significantly higher in the physiological saline treatment group compared to the OT treatment group.

**Conclusion:**

OT reduces diabetic retinopathy progression, particularly when administered intravitreally. To our knowledge, this is the first attempt to investigate the impact of OT on diabetic retinopathy and may provide a new area for further research.

## Introduction

Diabetes mellitus is a progressive disease that afflicts over 230 million people worldwide. Diabetes affects both microvascular and macrovascular structures throughout the body and consequently can cause retinopathy, neuropathy, and nephropathy. Diabetic retinopathy (DR) is the leading cause of preventable blindness. Sustained hyperglycemia causes the blood vessels to swell and leak fluid, resulting in damage to the microvascular structure of the retina. This can result in retinal ischemia that leads to vascular endothelial growth factor (VEGF) secretion and the growth of immature, fragile new vessels. These new vessels lead to neovascularization and proliferative DR and result in macular edema, vitreous hemorrhages, and tractional retinal detachment.^1,2,3,4,5^

Oxytocin (OT) is nonapeptide synthesized in the supraoptic and paraventricular nuclei of the hypothalamus. OT establishes its effects through the OT receptor, a G protein-coupled receptor. It stimulates uterine contractions at parturition, myoepithelial cell contraction in mammalian glands for milk ejection, and also has vasoconstrictor or vasodilator effects on different vascular beds.^6,7,8^ Recent studies have also reported the anti-inflammatory and anti-oxidant effects of OT.^9,10^ OT receptors have been found in cone photoreceptors and retinal pigment epithelium. OT exerts its effects by increasing intracellular levels of Ca^+2^, which facilitates smooth muscle contraction, nitric oxide synthesis, prostaglandin production, activation of the MAP-kinase cascade, and protein synthesis.^11,12^

In view of these previous studies and observations, we aimed to detect the effect of intravitreal and intraperitoneal administration of OT in the retina of streptozotocin (STZ)-induced diabetic rats.

## Materials and Methods

### Animals

In this study, 24 adult male and female Sprague Dawley rats weighing 200–250 g were used. Animals were fed ad libitum and housed in pairs in steel cages having a temperature-controlled environment (22±2 ^o^C) with 12-hour light/dark cycles. The experimental procedures were approved by the Committee for Animal Research of Ege University. All animal studies strictly conformed to the Committee on Animal Research and Ethics guidelines. All chemicals were obtained from Sigma-Aldrich Inc. unless otherwise noted.

### Experimental Protocol

Diabetes was induced by a single intraperitoneal injection of streptozocin (STZ) (Sigma-Aldrich, Inc., Saint Louis, MO) (60 mg/kg in 0.9% NaCl, adjusted to a pH 4.0 with 0.2 M sodium citrate). Diabetes was verified after 48 h by evaluating blood glucose levels with the use of glucose oxidase reagent strips (Boehringer Mannheim, Indianapolis). Rats with blood glucose levels of 260 mg/dl (14.4 mmol/L) and higher were included in this study as diabetic rats.

The 24 rats were equally divided into 4 groups as follows: intravitreal physiological saline group, intravitreal OT group, intraperitoneal physiological saline group, and intraperitoneal OT group. The rats in the intraperitoneal groups were treated daily with either 1 mL physiological saline or 100 IU/kg OT intraperitoneally; the rats in the intravitreal groups received topical anesthesia with proparacaine hydrochloride followed by 0.01 mL physiological saline or 10 μU/μL OT in weekly intravitreal injections. Hamilton syringes fitted with 27-gauge needles were used for intraperitoneal injections while 31-gauge needles were used for intravitreal injections. After 4 weeks of treatment the rats were euthanized and blood samples were collected by cardiac puncture for enzyme-linked immunosorbent assay (ELISA) of plasma VEGF levels in the intraperitoneal treatment groups, then enucleation was performed in all groups.

### Immunohistochemistry

Cross-sections 2 μm in thickness were taken with a microtome (Leica MR 2145) from paraformaldehyde-fixed paraffin-embedded eye tissues, floated in a sterile bath, placed onto poly-L-Lysine-coated glass slides, and dried at room temperature. After overnight incubation at 60 °C, the slides were dewaxed in xylene for 30 min, rehydrated through a graded ethanol series (100%, 95%, 80%, and 70%, sequentially), washed in distilled H_2_O and PBS for 10 min, treated with 2% trypsin containing 50 mM Tris buffer (pH 7.5) at 37 °C for 15 min, and then washed again with PBS. Sections were delineated with a Dako pen (Dako, Glostrup, Denmark), incubated in 3% H_2_O_2_ solution for 15 min to inhibit endogenous peroxidase activity, and washed with PBS. The slides were incubated with VEGF primary antibody at 57 °C followed by washing with PBS. Afterwards, a biotinylated secondary IgG antibody was applied and washed with PBS before incubating with the streptavidin-peroxidase conjugate (Histostain Plus, Invitrogen, Camarillo, CA, USA) for 30 min to visualize the immunostaining. The whole procedure was finished after counterstaining the sections with Mayer’s hematoxylin (Sigma Chemical Co., St. Louis, MO, USA). All sections were examined and photographed with an Olympus C-5050 digital camera mounted on an Olympus BX51 microscope (Olympus Corp., Tokyo, Japan).

### Outer Nuclear Layer (ONL) Measurements

All sections were photographed and measured with the same Olympus C-5050 digital camera mounted on an Olympus BX51 microscope. The mean ONL thickness in the physiological saline groups was accepted as 100%.

### Measurement of Plasma VEGF Levels

Plasma VEGF levels were measured using a commercially available ELISA kit according to the manufacturer’s instructions (RayBiotech, Inc., GA, USA). VEGF levels were expressed in pg/mL. The detection limit was less than 2 pg/mL, and intra-assay and interassay coefficients of variation were less than 10%.

### Statistical Analysis

Data analyses were performed using SPSS version 15.0 for Windows (SPSS Inc., Chicago, IL). The groups of parametric variables were compared using Student’s t-test. The groups of non-parametric variables were compared with the Mann-Whitney U test. The results were reported as mean ± standard error of mean. A value of p<0.05 was accepted as statistically significant.

## Results

VEGF protein expression was examined by immunohistochemistry and ONL thickness was measured. The expression was scored as follows: 0 represented no expression while 1, 2, and 3 represented expressions of 0-24%, 25-49%, 50-74%, and >75%. All comparisons of ONL measurements and staining intensities were carried out at X40 magnification from 10 different sections.

### ONL Measurements

**Figure 1:**
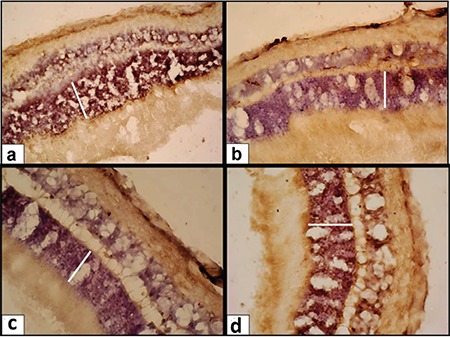
Outer nuclear layer of the a) intraperitoneal physiologic saline group, b) intravitreal physiologic saline group, c) intraperitoneal oxytocin group, and d) intravitreal oxytocin group

Figure 1 represents the alterations in retinal ONL thickness in the study groups. Results from the comparison of ONL thickness between groups is shown in Table 1. Morphometric analysis of the rat retinal cross-sections showed that intravitreal and intraperitoneal OT significantly increased ONL thickness compared to physiological saline-treated groups (23% and 25%, respectively, p<0.001).

### VEGF Protein Expression

**Figure 2:**
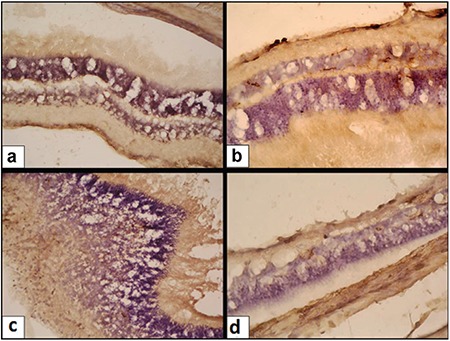
Vascular endothelial growth factor immunostaining in the a) intraperitoneal physiologic saline group, b) intravitreal physiologic saline group, c) intraperitoneal oxytocin group, and d) intravitreal oxytocin group

Figure 2 demonstrates VEGF protein expression in the rat retina after cessation of treatment. The mean score for VEGF expression in the intraperitoneal physiological saline group was 1.6±0.2, which decreased significantly to 0.3±0.2 in the intraperitoneal OT group. In the intravitreal treatment groups, the VEGF expression score was 1.3±0.3 in the physiological saline group, which decreased significantly to 0.3±0.2 in the OT group.

### Plasma VEGF Levels

Plasma VEGF level was 161.29±47.36 pg/mL (range: 115.75-225.25) in the intraperitoneal saline group and 76.74±14.15 pg/mL (range: 25.50-142.75) in the intraperitoneal OT group. There was a statistically significant difference between the groups.

## Discussion

To the best of our knowledge, this is the first report describing the effects of OT on the retina of diabetic rats. The study revealed that OT has protective effects on diabetic rat retina, as evidenced by reduced VEGF protein expression and plasma VEGF levels, as well as prevention of outer nuclear layer thinning.

As the pathogenesis of DR is better understood, novel treatment options are likely to become available. Postulated mechanisms of DR are hyperglycemia, accumulation of advanced glycation end-products (AGEs), activation of protein kinase C, oxidative stress, and inflammation. Chronic hyperglycemia results in the production of reactive oxygen species, and low-grade inflammation. This induces apoptosis of the retinal pigment epithelium and progression of DR. Hyperglycemia also causes accumulation of AGEs beneath the endothelial layer and changes the vascular structure, increases vascular stiffness, and initiates intracellular signaling pathways that lead to increased oxidative stress and inflammation. Oxidative stress is considered to be the most common mechanism in the etiology of DR. Damage or dysfunction due to oxidative stress can proceed even after glycemic control. Inflammation also plays an important role in the progression of DR and its complications. Therefore, new treatment strategies should be developed that target oxidative stress and inflammation.^1,13,14,15,16,17,18,19,20^

OT is nonapeptide and its receptors have been identified in different tissues including kidney, heart, pancreas, adipocytes, and thymus. The role of OT in immune and inflammatory modulation is well defined and attributed to the activation of its receptors.^6,7,8,21^ Based on these studies, we hypothesized that OT may act as an antioxidant and anti-inflammatory agent and therefore serve as a therapeutic agent in DR. Cone photoreceptors and the retinal pigment epithelium have OT receptors. Activation of those receptors causes an increase in intracellular Ca^+2^ level and downstream activation of phospholipase C and phosphatidylinositol 4,5-bisphosphate.^13,14^

In diabetic patients, sustained hyperglycemia can cause dysregulation in retinal blood flow, loss of pericytes, basal membrane thickening, microaneurysms, capillary occlusion, and ischemia. Ischemia eventually leads to the production of VEGF that activates tyrosine kinase receptors, VEGFR-1 and VEGFR-2.^22^ VEGF is a growth factor and potent vasoactive cytokine that promotes angiogenesis, breakdown of the blood-retinal barrier, and induces endothelial cell growth and neovascularization.^23,24^ Matsuoka et al.^25^ reported that expression of VEGF in diabetic retinas was significantly increased while Funatsu et al.^26^ suggested that VEGF levels in vitreous and plasma were elevated in diabetic patients with retinopathy when compared to normal subjects and diabetic patients without retinopathy. Consistent with this opinion, Aiello et al.^27^ found that VEGF concentration was elevated in the ocular fluids of patients with retinal ischemia. The present study showed that VEGF expression in retina and plasma VEGF levels were elevated, but with OT treatment, the expression and plasma levels were significantly decreased.

Thinning of the outer nuclear layer has been reported in diabetic rats. A number of studies have implicated apoptosis for the thinning effect.^28,29,30^ In the present study, in accordance with published reports, administration of OT was found to prevent the thinning of the ONL in STZ-induced diabetic rats when compared to the physiological saline group.

## Conclusion

In conclusion, the treatment of STZ-induced diabetic rats with OT was effective in mitigating retinal degeneration. The significant reduction in VEGF expression and plasma VEGF levels and the protective effect against retinal thinning suggest that OT may be an alternative treatment in diabetic retinopathy. The beneficial effects of OT in diabetic retinal degeneration might be through its anti-oxidative and anti-inflammatory effects. To our knowledge this is the first report about the effect of OT on the retina of diabetic rats, and this subject needs to be explored with further studies.

## Figures and Tables

**Table 1:**
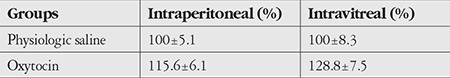
Comparison of outer nuclear layer thickness (x40 magnification)
